# Germinal center–dependent and –independent memory B cells produced throughout the immune response

**DOI:** 10.1084/jem.20202489

**Published:** 2021-06-09

**Authors:** Charlotte Viant, Tobias Wirthmiller, Mohamed A. ElTanbouly, Spencer T. Chen, Ervin E. Kara, Melissa Cipolla, Victor Ramos, Thiago Y. Oliveira, Leonidas Stamatatos, Michel C. Nussenzweig

**Affiliations:** 1 Laboratory of Molecular Immunology, The Rockefeller University, New York, NY; 2 Vaccine and Infectious Disease Division, Fred Hutchinson Cancer Research Center, Seattle, WA; 3 Department of Global Health, University of Washington, Seattle, WA; 4 Howard Hughes Medical Institute, The Rockefeller University, New York, NY

## Abstract

Memory B cells comprise a heterogenous group of cells that differ in origin and phenotype. During the early phases of the immune response, activated B cells can differentiate into IgM-expressing memory cells, short-lived plasma cells, or seed germinal centers (GCs). The memory compartment is subsequently enriched by B cells that have been through several rounds of division and selection in the GC. Here, we report on the use of an unbiased lineage-tracking approach to explore the origins and properties of memory B cell subsets in mice with an intact immune system. We find that activated B cells continue to differentiate into memory B cells throughout the immune response. When defined on the basis of their origins, the memory B cells originating from activated B cells or GCs differ in isotype and overall gene expression, somatic hypermutation, and their affinity for antigen.

## Teaser

Memory B cells comprise a heterogenous group of cells. Viant et al. report on the use of an unbiased lineage-tracking approach to explore the origins and properties of memory B cell subsets in mice with an intact immune system.

## Introduction

Humoral memory responses are essential for immunity to pathogens and for effective vaccination. As suggested by Burnet (1959) in his clonal selection theory, formation of memory involves significant clonal expansion and diversification. The cellular components that produce humoral memory are found in two different compartments: long-lived plasma cells and memory B cells (Goodnow et al., 2010; Harwood and Batista, 2010; Victora and Nussenzweig, 2012; Vinuesa et al., 2016; Akkaya et al., 2020; Lau and Brink, 2020; Laidlaw and Cyster, 2021). As might be expected, long-lived plasma cells producing high-affinity antibodies develop from precursors that are selected based on their affinity for antigen (Smith et al., 2000; Phan et al., 2006; Kräutler et al., 2017). In contrast, memory B cells show a broad range of different affinities, including cells producing antibodies that have no measurable ability to bind to the cognate antigen (Viant et al., 2020; Wong et al., 2020).

Two subsets of memory B cells develop during the immune response. The first subset is thought to be derived from activated B cells that are germinal center (GC) independent because they arise early, at the time that GCs are just beginning to coalesce (Pape et al., 2011; Weisel et al., 2016). The second group of memory B cells is derived from GCs (Laidlaw et al., 2020; Laidlaw et al., 2017; Shinnakasu et al., 2016; Suan et al., 2017; Wang et al., 2017). Under physiological circumstances, memory formation involves T–B cell interactions and Bcl6 expression. However, neither is absolutely required (Kaji et al., 2012; Matsumoto et al., 1996; Obukhanych and Nussenzweig, 2006; Toyama et al., 2002).

Elegant single-cell transfer experiments showed that GC-independent memory B cells develop from activated B cells (Act-Bmem cells) beginning sometime before day 3 after immunization (Taylor et al., 2012). These cells make a major contribution to the memory compartment during the early phase of the immune response (Blink et al., 2005; Pape et al., 2011; Taylor et al., 2012; Weisel et al., 2016). The initial burst of memory formation from activated B cells is thought to be supplanted later in the immune response by GC-dependent memory B cells (GC-Bmem cells; Laidlaw et al., 2020; Laidlaw et al., 2017; Shinnakasu et al., 2016; Suan et al., 2017; Wang et al., 2017). Selection into the GC-Bmem cell compartment is associated with increased CCR6 (Suan et al., 2017), Ephrin-B1 (Laidlaw et al., 2017), Bach-2 (Shinnakasu et al., 2016), Tle3, and Hhex (Laidlaw et al., 2020) expression; decreased Bcl6 (Wang et al., 2017) expression; lower mTORC1 activity (Inoue et al., 2021); and cell-cycle arrest (G0 phase; Wang et al., 2017). GC-Bmem cells tend to have lower antigen-binding affinity than B cells that remain in the GC (Shinnakasu et al., 2016; Suan et al., 2017; Viant et al., 2020).

Memory B cells are currently identified by specific antigen binding (Shinnakasu et al., 2016; Suan et al., 2017; Viant et al., 2020), differential expression of antibody isotypes (Dogan et al., 2009; Pape et al., 2011), and surface markers such as CD80 and PDL2 (Tomayko et al., 2010; Zuccarino-Catania et al., 2014) and not on the basis of their origins. Here, we report on the use of an unbiased lineage-tracking approach that does not rely on antigen binding, class-switch recombination, or cell surface marker expression to explore the origins and properties of memory B cells during polyclonal immune responses to an HIV-1 antigen, TM4-Core (Dosenovic et al., 2015).

## Results

### Identification of two memory B cell populations

In the early phases of the immune response, memory B cells are derived primarily from precursors that undergo only a small number of divisions and appear to be GC independent (Blink et al., 2005; Pape et al., 2011; Taylor et al., 2012; Weisel et al., 2016). At later time points, memory cells are thought to originate from the GC (Laidlaw et al., 2020; Laidlaw et al., 2017; Shinnakasu et al., 2016; Suan et al., 2017; Wang et al., 2017). To further characterize the contribution of these two populations to the memory compartment during the immune response, we developed a lineage-tracking approach. Memory cells derived from precursors with limited or extensive cell division are identified by combining an H2B-mCherry reporter (*Vav*^Tg^ *Col1a*^mCherry/+^) and the *S1pr2*^CreERT2/+^ *R26*^ZSGreen/+^ indicator mice (double-reporter mice; Gitlin et al., 2014; Madisen et al., 2010; Shinnakasu et al., 2016).

**Figure S1.**
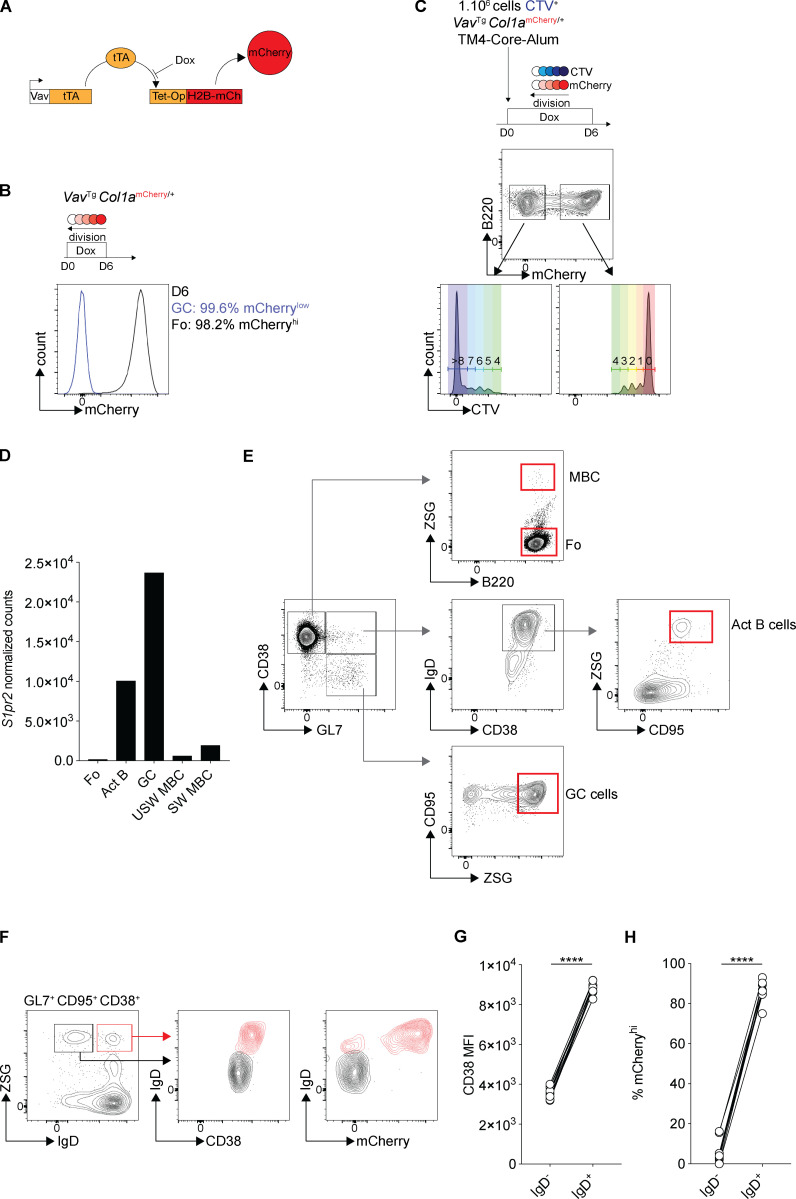
**Identification of two memory B cell populations. **Related to Fig. 1. **(A)** Schematic representation of the Vav-tTA and Tet-Op-H2B-mCh transgenes that were combined (tTA–H2B–mCh) to label B cells with H2B–mCherry to measure cell division in the B cells after doxycycline (Dox) treatment. **(B)** Representative histogram showing the mCherry expression in GC cells (blue) and follicular (Fo) B cells (black) 6 d after doxycycline administration to *Vav*^Tg^ *Col1a*^mCherry/+^ mice (three independent experiments). **(C)** B cells from CD45.2 *Vav*^Tg^ *Col1a*^mCherry/+^ mice were stained with CellTrace Violet (CTV) in vitro and transferred into a CD45.1 host immunized with HIV-1 TM4-Core the same day. 6 d after immunization, the GCs were analyzed by flow cytometry for mCherry and CTV dilution (three independent experiments). **(D)** Graphs show the level of expression of *S1pr2* in follicular (Fo) B cells, activated B cells (Act B), GC cells, IgM, or switched isotype–expressing memory B cells (MBC) obtained by RNA-seq. **(E)** Gating strategy for ZSGreen^−^ (ZSG^−^) Fo B cells (CD38^+^, GL7^−^, ZSG^−^), ZSG^+^ Act B cells (CD38^+^, GL7^+^, IgD^+^, CD95^+^, ZSG^+^), ZSG^+^ GC cells (CD38^−^, GL7^+^, CD95^+^, ZSG^+^), and ZSG^+^ MBCs (CD38^+^, GL7^−^, ZSG^+^). **(F–H)** Representative flow cytometry profiles and graphs showing the percentage of mCherry^low^ and CD38 median fluorescence intensity (MFI) among IgD^−^ (black) and IgD^+^ (red) activated B cells (CD38^+^, CD95^+^, GL7^+^, ZSG^+^). (Each dot represents one mouse, three independent experiments, *n* = 10–11.) ****, P ≤ 0.0001 by paired *t* test. D, day; USW MBC, unswitch memory B cells IgD^+^ or IgM^+^; SW MBC, switch memory B cells IgD^−^ IgM^−^.

H2B-mCherry mice carry a constitutively expressed indicator gene under the control of a tetracycline transactivator protein. Administration of doxycycline represses H2B-mCherry synthesis, resulting in dilution of the indicator in direct proportion to cell division (Fig. S1 A; Gitlin et al., 2014). For example, whereas follicular B cells, which divide only infrequently, remain mCherry^hi^, after doxycycline administration, contemporaneous GC cells dilute the indicator completely (Fig. S1 B). When compared directly with a different proliferation tracker, CellTrace Violet, mCherry^low^ B cells divided at least four times, and mCherry^hi^ cells divided zero to four times (Fig. S1 C). Tamoxifen administration to *S1pr2*^CreERT2/+^ *R26*^ZSGreen/+^ mice permanently labels activated or GC B cells and their progeny, but not follicular origin or memory B cells, by excision of a stop cassette in the ROSA26-lox-stop-lox-ZSGreen reporter gene (Fig. S1, D and E). GC cells that are mCherry^low^ express GL7, CD95, and S1pr2 (Fig. S1, D and E), while recently activated B cells that are mCherry^hi^ still express IgD and CD38 follicular B cell markers (Fig. S1, D and E). A second population of what appear to be more differentiated IgD^−^-activated B cells are mCherry^low^ and show decreased CD38 expression (Fig. S1, F–H). This intermediate group appears to be in the GC pathway and was omitted from further consideration.

**Figure 1.**
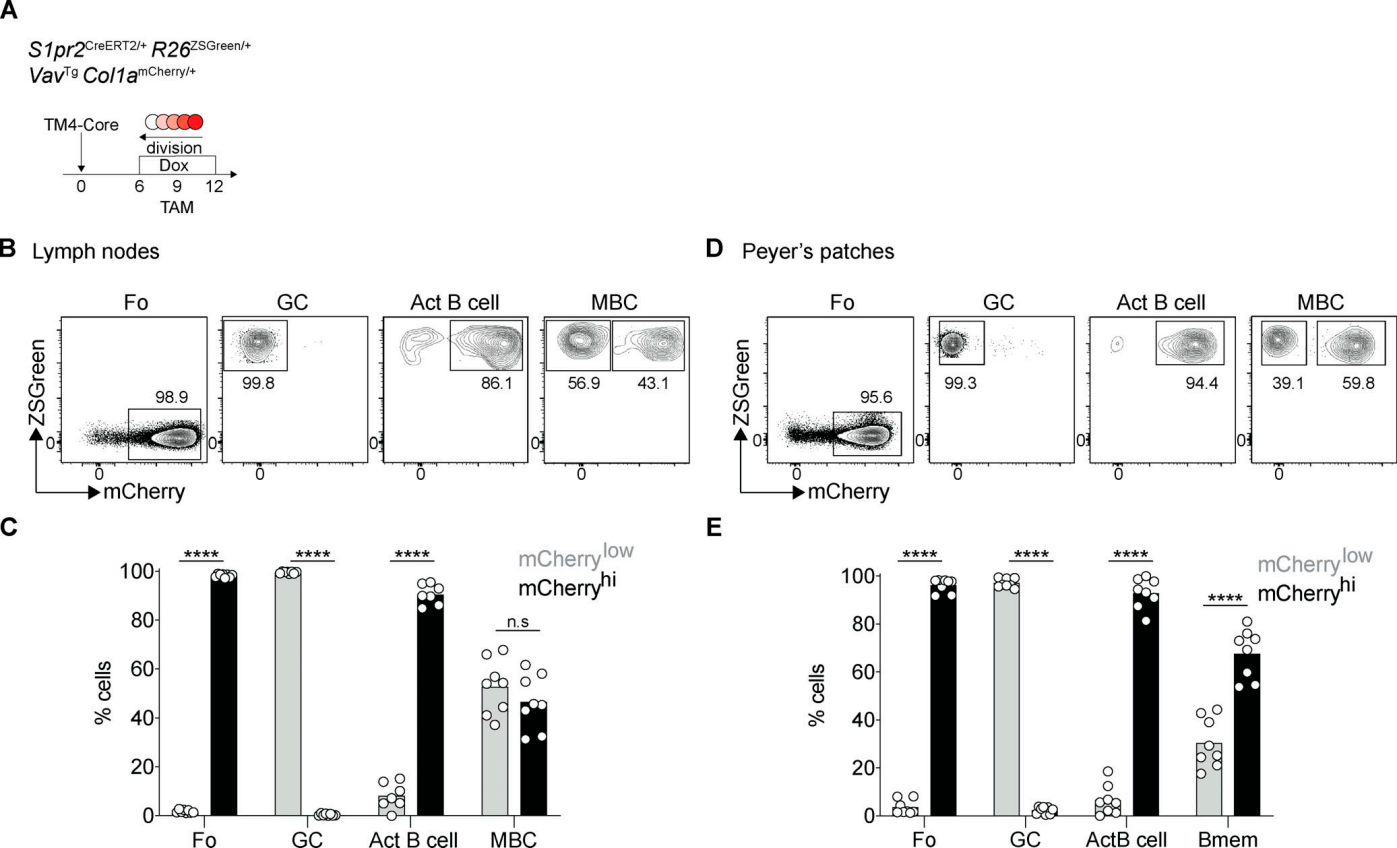
**Identification of two memory B cell populations.** **(A)** Schematic representation of the experiment: *S1pr2*^CreERT2/+^ *R26*^ZSGreen/+^ *Vav*^Tg^ *Col1a*^mCherry/+^ mice immunized with HIV-1 TM4-Core on day 0, doxycycline (Dox) administered on days 6–12, and tamoxifen (TAM) on day 9. Analysis was performed on day 12. **(B and C)** Representative flow cytometry profiles and graph showing the percentage of mCherry^low^ and mCherry^hi^ cells among follicular (Fo), GC, activated B cells (Act B cell), and memory B cells (MBC) from the lymph nodes. (Each dot represents one mouse, three independent experiments, *n* = 8–11.) ****, P ≤ 0.0001 by two-way ANOVA. **(D and E)** As in B and C in the Peyer’s patches. (Each dot represents one mouse, three experiments, *n* = 8.) ****, P ≤ 0.0001 by one-way ANOVA.

To test the combination of fate mapping and cell division tracking in double-reporter mice, we immunized the mice with the HIV-1 envelope-based immunogen TM4-Core (Dosenovic et al., 2015); administered doxycycline and tamoxifen starting on days 6 and 9, respectively; and analyzed on day 12 after immunization (Fig. 1, A–C). Under these conditions, nearly all ZSGreen^+^ GC cells were mCherry^low^ (Fig. 1, B and C), and therefore, GC-derived memory B cells should also be similarly mCherry^low^. In contrast, contemporaneously labeled activated B cells are 88% mCherry^hi^, indicating that the great majority of memory B cells derived from this compartment should be mCherry^hi^ (Fig. 1, B and C). On day 12 after immunization, memory B cells were heterogeneous, with 47% mCherry^hi^ and 53% mCherry^low^ (Fig. 1, B and C). Thus, the lineage-tracking system identifies two populations of ZSGreen^+^ memory B cells: mCherry^low^ memory B cells, which predominantly originate from highly proliferative GC precursors, GC-Bmem cells, and a second group derived from the activated B cell compartment, Act-Bmem cells. The same two memory B cell populations are also present in the Peyer’s patches in the absence of immunization (Fig. 1, D and E).

### Activated B cells continue to differentiate into memory B cells throughout the immune response

**Figure 2.**
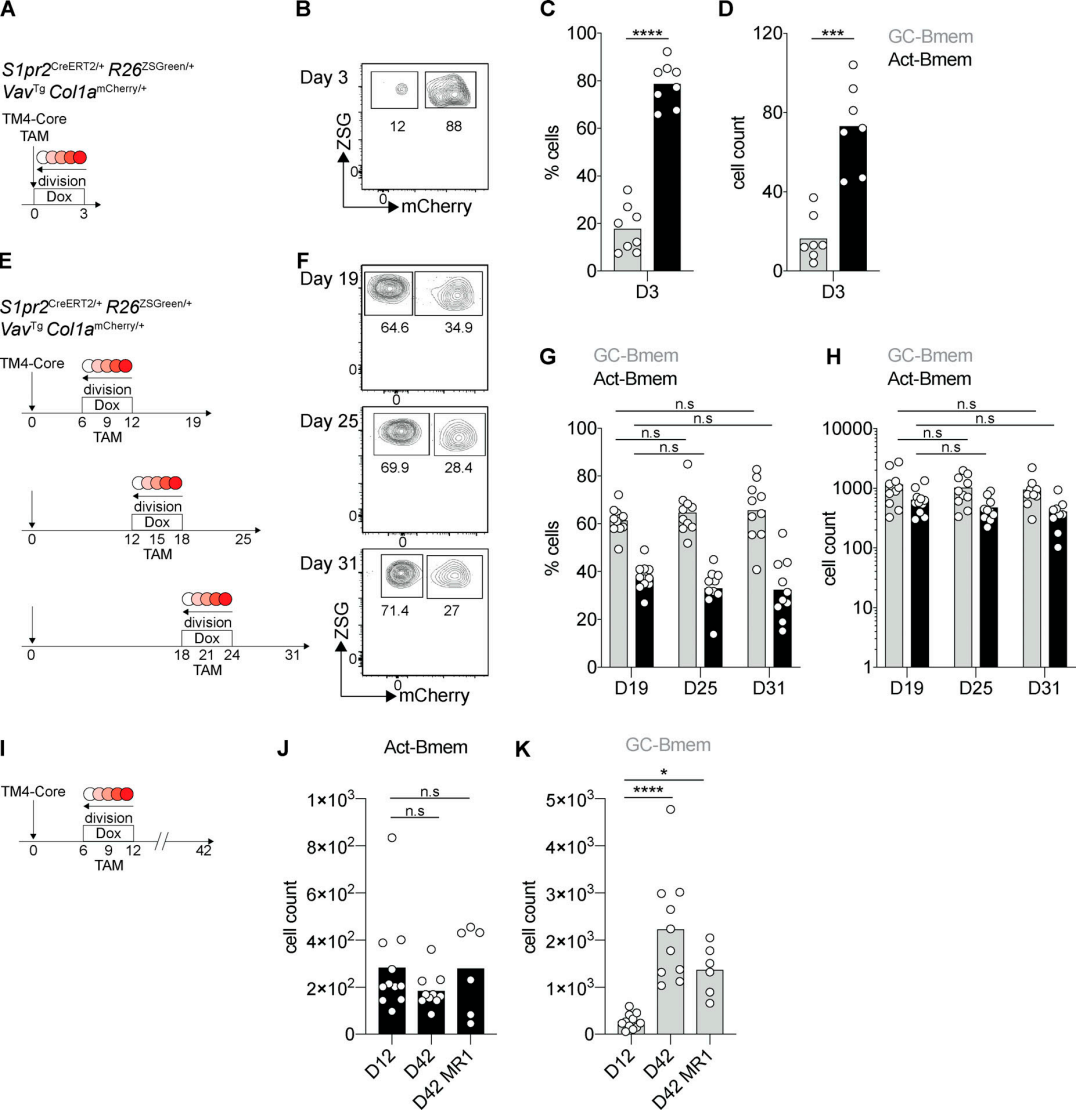
**Activated B cells and GC B cells produce memory B cells throughout the immune response.** **(A)** Schematic representation of the experiment: *S1pr2*^CreERT2/+^ *R26*^ZSGreen/+^ *Vav*^Tg^ *Col1a*^mCherry/+^ mice were immunized with HIV-1 TM4-Core and treated with tamoxifen (TAM) and doxycycline (Dox) on the same day. Analysis was performed 3 d after immunization. **(B)** Representative flow cytometry profiles showing mCherry expression by ZSGreen^+^ (ZSG^+^) memory B cells at day 3 (D3; A). **(C and D)** Graphs show summary of data from three independent experiments described in A, showing the percentage (C) and the number of cells (D). (Each dot represents one mouse, three independent experiments, *n* = 8.) ***, P ≤ 0.001; ****, P ≤ 0.0001 by paired *t* test. **(E)** Schematic representation of the experiment: three groups of *S1pr2*^CreERT2/+^ *R26*^ZSGreen/+^ *Vav*^Tg^ *Col1a*^mCherry/+^ mice were immunized with HIV-1 TM4-Core on day 0; doxycycline was administered on the following days: group 1, day 6–12; group 2, day 12–18; group 3, day 18–24. Tamoxifen was administered on day 9 for group 1, day 15 for group 2, and day 21 for group 3. Analysis was performed 10 d after tamoxifen administration. **(F)** Representative flow cytometry profiles showing mCherry expression by ZSG^+^ memory B cells at the three time points described in A. **(G and H)** Graphs show summary of data from three independent experiments described in B at each time point, showing the percentage (C) and the number of cells (D). (Each dot represents one mouse, three independent experiments, *n* = 9–11, two-way ANOVA.) **(I)** Schematic representation of the experiment: *S1pr2*^CreERT2/+^ *R26*^ZSGreen/+^ *Vav*^Tg^ *Col1a*^mCherry/+^ mice were immunized with HIV-1 TM4-Core on day 0, and doxycycline was administered on day 6–12 and tamoxifen on day 9. One group of mice was analyzed on day 12; the other groups were analyzed on day 42 with or without MR1 (anti-CD40L, i.v. injection) every 3 d starting at day 20. **(J and K)** Graphs show the number of Act-Bmem cells (J) or GC-Bmem cells (K) at day 12 (D12) or 42 (D42) with or without injection of MR1. (Each dot represents one mouse, two to three independent experiments, *n* = 6–11.) *, P ≤ 0.05; ****, P ≤ 0.0001 by two-way ANOVA.

A majority of the B cells entering the memory compartment in the early phase of the immune response, before GC formation, are derived from the activated B cell pool (Fig. 2, A–D; Taylor et al., 2012). However, the contribution of activated B cells to the memory compartment in the later stages of the immune response has not been determined. To document the ongoing contribution of Act-Bmem cells to the memory compartment in later stages of the immune response, we immunized mice with HIV-1 TM4-Core and administered doxycycline and then tamoxifen on days 6 and 9, 12 and 15, or 18 and 21 after immunization, respectively (Fig. 2 E). Analysis on day 10 after tamoxifen administration (19, 25, or 31 d after immunization) showed that the relative proportion of emerging Act-Bmem and GC-Bmem cells was similar throughout the observation period (Fig. 2, F–H). We conclude that GCs and activated B cells produce memory B cells throughout the immune response.

**Figure S2.**
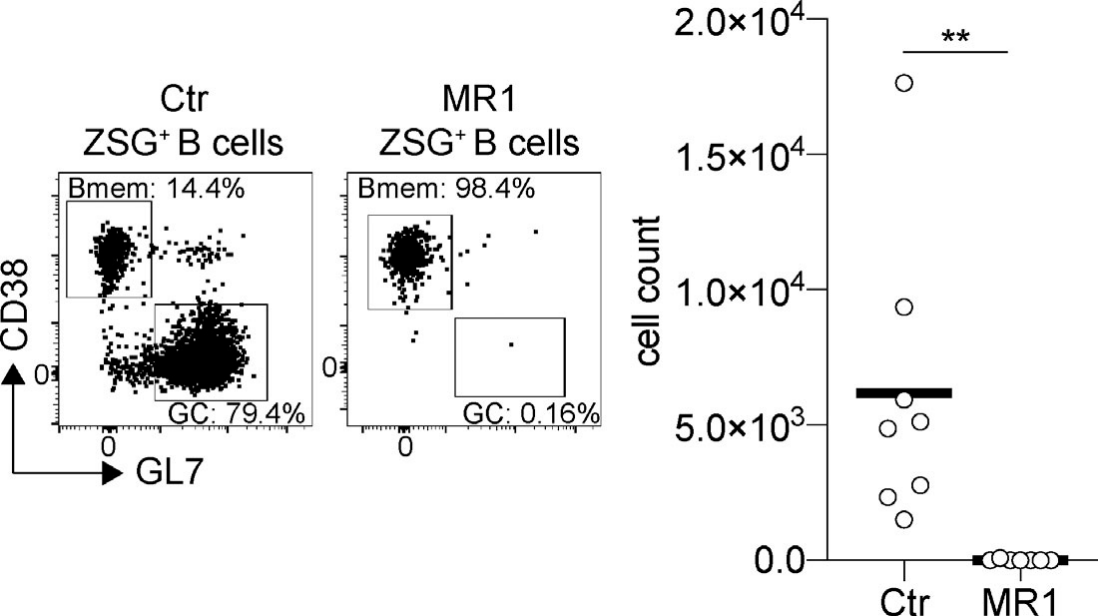
**ZSGreen^+^ GC cell depletion. **Related to Fig. 2. Representative flow cytometry profiles and graph showing the percentage and the number of ZSGreen^+^ (ZSG^+^) GC cells analyzed as in Fig. 2 I at day 42 with or without CD40L blocking antibody injections. (Each dot represents one mouse, two independent experiments, *n* = 7–8.) **, P ≤ 0.01 unpaired *t* test. Ctr, control.

To determine whether the two types of emerging memory cells are retained at later time points, we labeled with tamoxifen on day 9 and assayed on days 12 and 42 (Fig. 2, I–K). Because the GC remains active throughout, we inhibited that GC reaction by repeatedly injecting with a CD40L-blocking antibody beginning on day 20 after immunization (MR1; Fig. S2). The number of Act-Bmem cells remained stable over the observation period, and GC-Bmem cells accumulated irrespective of CD40L antibody treatment (Fig. 2, I–K; and Fig. S2). Thus, Act-Bmem and GC-Bmem cells emerging early in the response are present in the draining lymph nodes 42 d after immunization.

### Gene expression profiles of GC-Bmem and Act-Bmem cells are distinct

**Figure S3.**
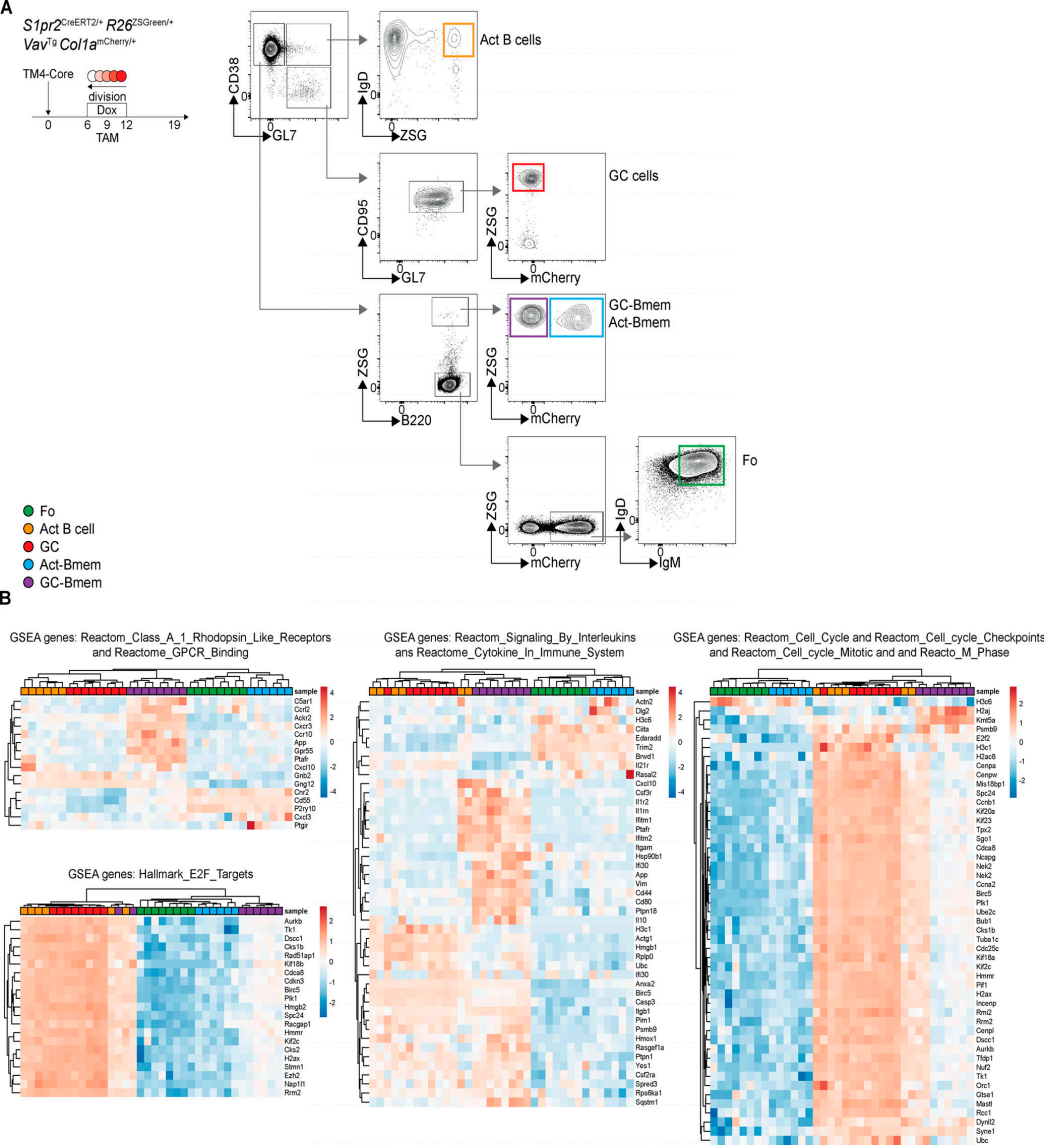
**Gene expression profiles of GC-Bmem and Act-Bmem cells are distinct. **Related to Fig. 3. **(A)** Gating strategy used for purifying ZSGreen^−^ (ZSG^−^) mCherry^hi^ follicular (Fo) B cells, ZSG^+^ Act B cells, ZSG^+^ mCherry^low^ GC cells, ZSG^+^ GC-Bmem cells, and ZSG^+^ Act-Bmem cells. **(B)** Heat map shows hierarchical clustering based on expression of 16 expressed genes in Reactome class A 1 Rhodopsin-like receptors and Reactome GPCR binding; 21 expressed genes in hallmark E2F targets; 45 expressed genes in Reactome signaling by interleukins and Reactome cytokine in immune system; and 49 expressed genes in Reactome cell cycle, Reactome cell cycle checkpoints, Reactome cell cycle mitotic, and Reactome M phase by Fo, GC, and activated B cells (Act B cell) and Act-Bmem and GC-Bmem cells. Dox, doxycycline; TAM, tamoxifen.

To determine whether the two memory B cell populations express different genetic programs, we compared purified populations of follicular B cells, activated B cells, GC B cells, Act-Bmem cells, and GC-Bmem cells by RNA sequencing (RNA-seq; Fig. S3 A). All five populations were purified from four independent groups of double-reporter mice that received doxycycline and tamoxifen on days 6–12 and 9, respectively, and were purified on day 19 after immunization with HIV-1 TM4-Core (Fig. S3 A).

**Figure 3.**
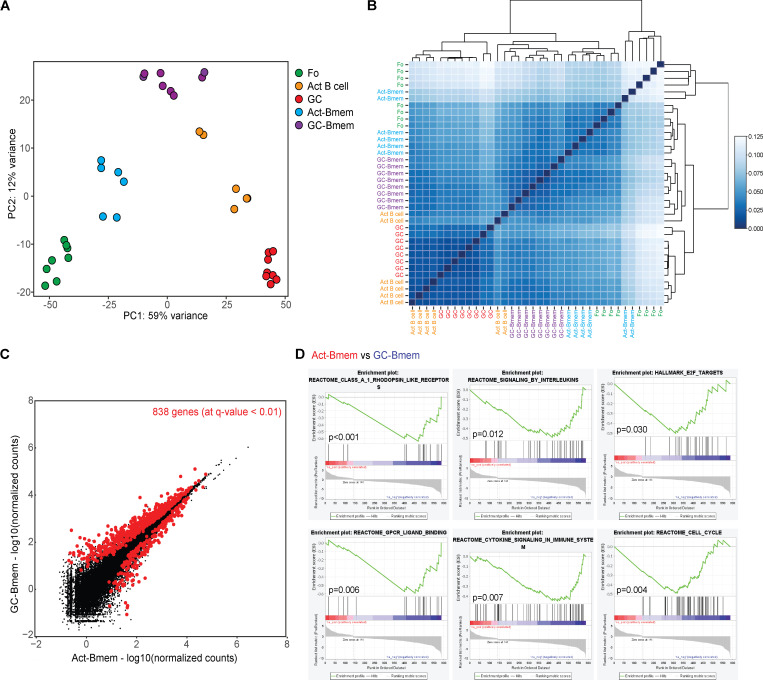
**Gene expression by GC-Bmem and Act-Bmem cells.** **(A and B)** Principal component (PC) analysis (A) and unsupervised hierarchical clustering (B) of follicular (Fo) B cells, GC B cells, activated B cells (Act B cell), Act-Bmem cells, and GC-Bmem cells. **(C)** Scatter plot shows the genes differentially expressed between GC-Bmem and Act-Bmem cells. 838 genes are significantly differentially expressed (red dots indicate q-value <0.01). **(D)** Graphical representation of GSEA and the rank-ordered gene lists found upregulated in GC-Bmem versus Act-Bmem cells in Reactome class A 1 Rhodopsin-like receptors (P < 0.001), Reactome GPCR ligand binding (P = 0.006), Reactome signaling by interleukins (P = 0.012), Reactome cytokine signaling in immune system (P = 0.007), hallmark E2F targets (P = 0.030), and Reactome cell cycle (P = 0.004).

Principal component analysis revealed five distinct populations that segregate independently (Fig. 3 A). Unsupervised hierarchical clustering revealed that the two memory populations were closely related to each other, which is consistent with the idea that both memory populations return to a resting state (Fig. 3 B). Despite their overall similarity in gene expression, GC-Bmem cells were more closely related to GC B cells, and Act-Bmem cells were most closely related to follicular origin B cells (Fig. 3 B). Although they are closely related, GC-Bmem and Act-Bmem cells differ in their expression of 838 genes (Fig. 3 C; q-value <0.01). Gene set enrichment analysis (GSEA) revealed significant increases in the following pathways in GC-Bmem compared with Act-Bmem cells: Class A 1 Rhodopsin-like receptors, GPCR ligand-binding pathway, interleukin and cytokine signaling, and cell-cycle and E2F pathways (Fig. 3 D and Fig. S2 B). Most of the genes in these pathways are also expressed in GC B cells but not in follicular B cells (Fig. S2 B).

**Figure 4.**
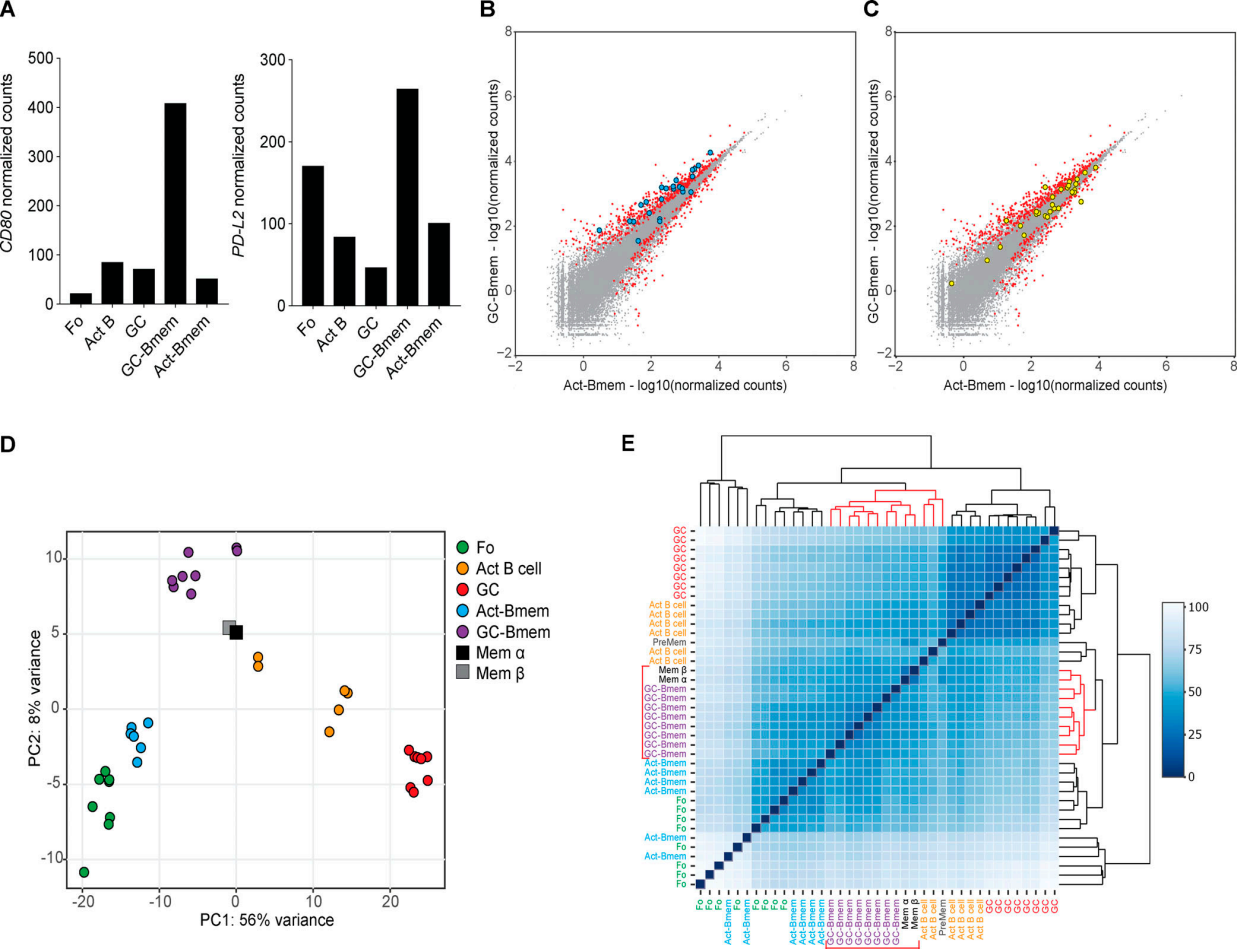
**Comparison of GC-Bmem and Act-Bmem cells with previously described memory B cell populations.** **(A)** Graphs show the level of expression of *Cd80* and *PD-L2* in follicular (Fo), activated B (Act B cell), and GC B cells; GC-Bmem cells; and Act-Bmem cells as determined by RNA-seq. **(B and C)** Scatter plot shows the gene expression differences between GC-Bmem and Act-Bmem cells as in Fig. 3 C. 24 genes known to be significantly more highly expressed in CD80^+^PD-L2^+^ than in CD80^−^PD-L2^−^ memory B cells are represented by blue circles (B). 30 genes known to be significantly more highly expressed in CD80^−^PD-L2^−^ than in CD80^+^PD-L2^+^ memory B cells are represented by yellow circles (C). **(D and E)** Principal component (PC) analysis (D) and unsupervised hierarchical clustering (E) of Fo, GC, and Act B cells; Act-Bmem cells; and GC-Bmem cells and the previously described Mem α and Mem β.

To investigate the relationship between GC-Bmem and Act-Bmem cells and previously identified memory B cell populations, we compared them to CD80^−^PD-L2^−^ and CD80^+^PD-L2^+^ memory B cells (Zuccarino-Catania et al., 2014). GC-Bmem cells express higher levels of CD80 and PD-L2 than other B cells (Fig. 4 A). Among 24 genes that are expressed at higher levels by CD80^+^PD-L2^+^ than CD80^−^PD-L2^−^ cells (Fig. 4 B), 13 are also differentially expressed by GC-Bmem cells (q-value <0.05; Fig. 4 B and Table S1). Thus, GC-Bmem cells are closely related to the CD80^+^PD-L2^+^ memory subset. However, Act-Bmem cells do not appear to overlap significantly with CD80^−^PD-L2^−^ at the transcriptional level. Of the 30 genes that are expressed at higher levels in CD80^−^PD-L2^−^ than CD80^+^PD-L2^+^ memory B cells, only three are coincidental in Act-Bmem cells (Fig. 4 C and Table S2). We also compared GC-Bmem and Act-Bmem cells to IgD^−^ Mem α and Mem β (Laidlaw et al., 2020). Principal component analysis and unsupervised hierarchical clustering revealed that both these populations are closely related to GC-Bmem cells (Fig. 4, D and E).

Thus, the gene expression profiles obtained from GC-Bmem and Act-Bmem cells indicate that the two cell types represent closely related, but distinct populations of memory B cells. GC-Bmem cells are most closely related to CD80^+^PD-L2^+^, Mem α and Mem β subpopulations, a finding that is consistent with the idea that they all originate in the GC.

### Somatic mutation and class-switch recombination in GC-Bmem and Act-Bmem cells

**Figure S4.**
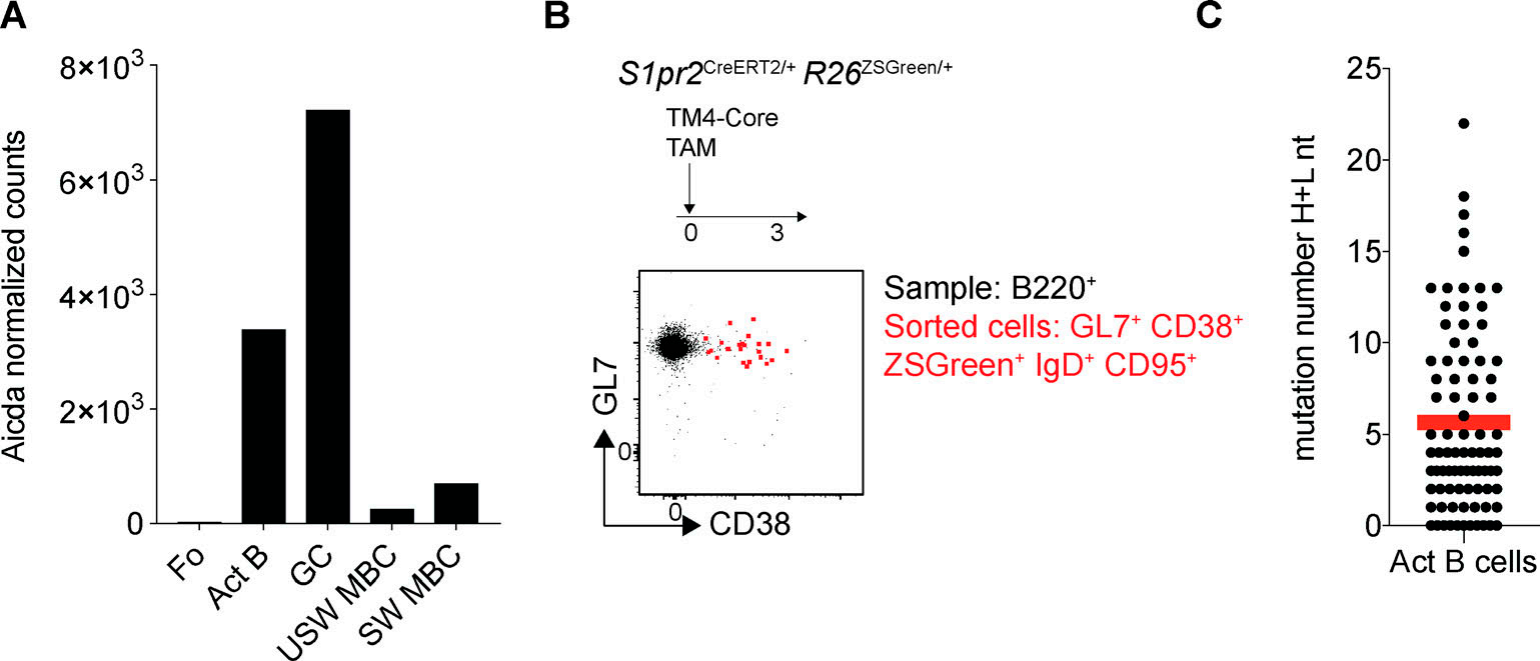
**Somatic mutation in activated B cells. **Related to Fig. 5. **(A)** Graphs show the level of expression of *ACIDA* in Fo B cells, activated B cells (Act B), GC cells, IgM, or switch isotype–expressing memory B cells (MBC) obtained by RNA-seq. **(B)** Representative flow cytometry profiles showing B220^+^ B cells 3 d after HIV-1 TM4-Core immunization and tamoxifen (TAM) administration and Act B (GL7^+^, CD38^+^, ZSGreen^+^, IgD^+^, CD95^+^) sorted from the same sample. **(C)** Graph showing the number of somatic mutations (nucleotides, IgH + IgL [H+L nt]) in the antibodies obtained from Act B 3 d after immunization (three independent experiments, *n* = 3–5 mice per group, each dot represents one antibody, *n* = 84 antibody genes sequenced).

**Figure 5.**
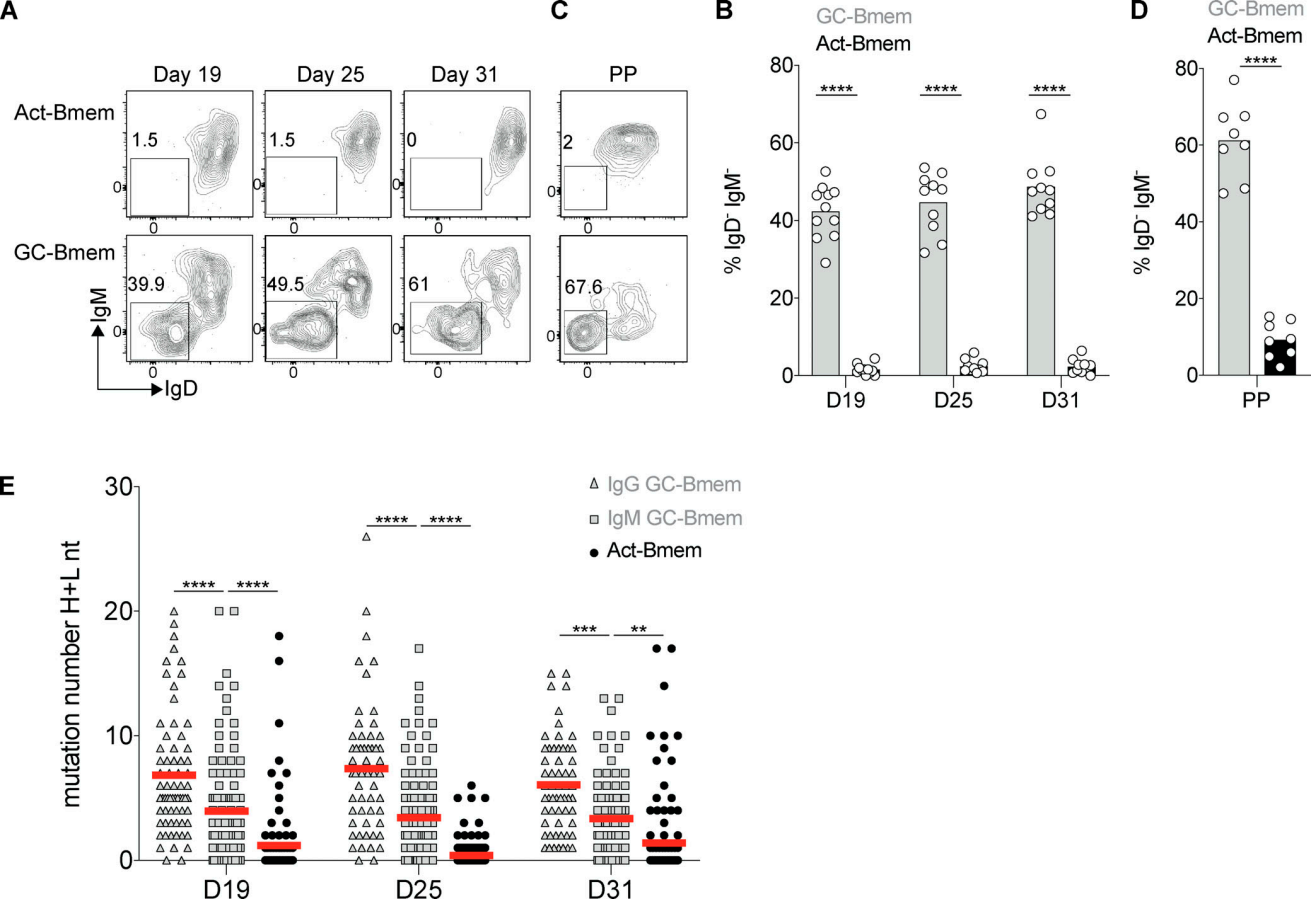
**Somatic mutation and class-switch recombination in GC-Bmem and Act-Bmem cells.** **(A and B)** Representative flow cytometry profiles (A) and summary of three independent experiments (B) showing the percentage of IgD^−^IgM^−^ among the GC-Bmem and Act-Bmem cells analyzed at the same time points as Fig. 2 A from the lymph nodes (Each dot represents one mouse, three independent experiments, *n* = 10–11.) ****, P ≤ 0.0001 by two-way ANOVA. **(C and D)** As in A and B in the Peyer’s patches (PP). (Each dot represents one mouse, three independent experiments, *n* = 8.) ****, P ≤ 0.0001 by *t* test. **(E)** Graph shows number of somatic mutations (nucleotides, IgH + IgL [H+L nt]) in the antibodies obtained from IgG^+^ GC-Bmem cells IgM^+^ GC-Bmem cells, and Act-Bmem cells, purified after immunization, tamoxifen gavage, and doxycycline administration, as in Fig. 2 A. The data represent three independent experiments with a combined total of 7–15 mice per group. (Each dot represents one antibody, *n* = 89–168 antibody genes sequenced in each group.) **, P ≤ 0.01; ***, P ≤ 0.001; ****, P ≤ 0.0001 by two-way ANOVA. D, day.

GC entry is not required for somatic hypermutation or class-switch recombination (Toellner et al., 2002; William et al., 2002; Roco et al., 2019). Activated B cells express *AICDA* (Fig. S4 A) and begin to mutate their antibody genes as early as 3 d after immunization, which is before GC formation (Fig. S4, B and C). Nearly half of the GC-Bmem cells, but only 1%–3% of contemporaneous Act-Bmem cells, had undergone class-switch recombination at the time points assayed (day 19: 42.3% vs. 1.6%, P < 0.0001; day 25: 44.6% vs. 2.4%, P < 0.0001; day 31: 48.6% vs. 2.3%, P < 0.0001; Fig. 5, A and B). Similar results were obtained by analyzing Peyer’s patches in the absence of immunization (Fig. 5, C and D). To examine the antibody genes expressed by B cells comprising the two memory compartments at the three time points described above, we purified single cells and sequenced their antibody genes (Fig. 2 A). Somatic mutations were significantly different between the two memory B cell populations, irrespective of the time they emerged during the immune response (Fig. 5 E). The number of mutations in GC-Bmem cells were significantly higher than in Act-Bmem cells at all time points, even when IgG-expressing cells were omitted from the analysis (Fig. 5 E). In addition, GC-Bmem cells expressing secondary antibody isotypes were significantly more mutated than GC-Bmem cells expressing IgM (Fig. 5 E). Thus, Act-Bmem cells that carry few somatic mutations and rarely express secondary isotypes enter the memory compartment throughout the immune response.

### Clonal origins of GC-Bmem and Act-Bmem cells

**Figure 6.**
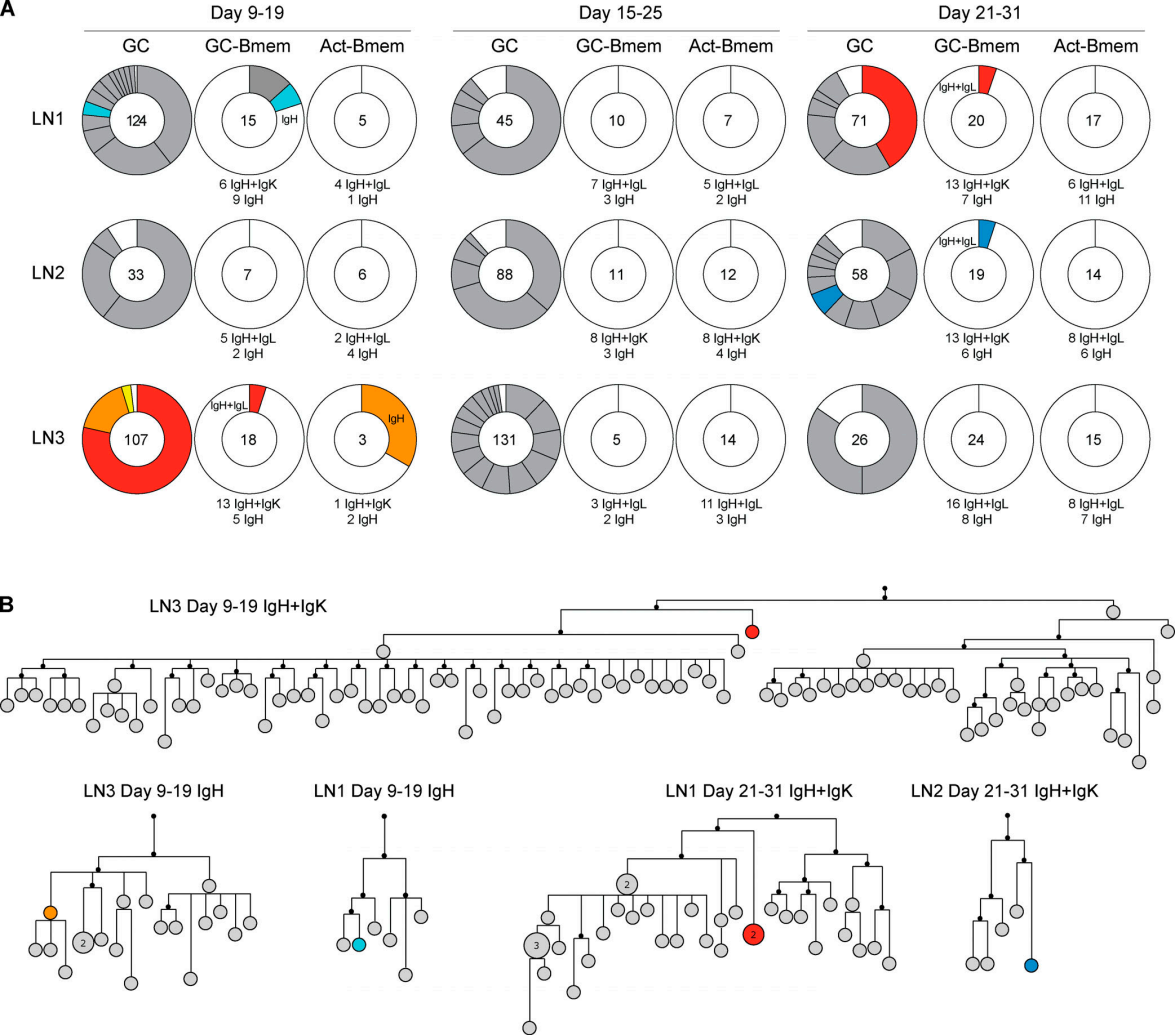
**Clonal origins of GC-Bmem and Act-Bmem cells.** **(A)** Pie charts show the clonal distribution of antibodies obtained from GC B cells, GC-Bmem cells, and Act-Bmem cells in single lymph nodes at the three time points described in Fig. 2 A. The number in the middle of the pie chart represents the number of IgH + IgL or IgH-alone antibody genes sequenced as indicated below each pie chart. Each slice is proportional to the number of clonal relatives. Colored slices indicate a common sequence found in different populations in the same lymph node as indicated in the pie chart. **(B)** Diagram shows phylogenetic relationships between shared antibody sequences obtained from GC (gray) and memory B (colors) cells in the clones identified in A.

To examine the relationship between the two memory populations and contemporaneous GC B cells, we examined the antibody sequences of B lymphocytes obtained from single lymph nodes at the three different time points described above (Figs. 2 A and 6 A). As expected, when all time points and lymph nodes are combined, 94% of all GC B cells were members of expanded clones that shared IgH and IgL (Fig. 6 A). Conversely, in the absence of antigen-binding selection, expanded clones were rarely found between the two memory B cell populations. Only 2.3% of the memory B cells produced in response to HIV-1 TM4-Core immunization shared antibody sequences with the expanded clones found in the contemporaneous GC (Fig. 6 A). Among the 93 Act-Bmem cells, we found only one example of an IgH sequence shared with a contemporaneously expanded GC B cell clone (days 9–19, LN3; Fig. 6 A). Among 129 GC-Bmem cells analyzed, 4 shared antibody sequences with contemporaneous GC B cells (days 9–19, LN1 and LN3; days 21–31, LN1 and LN2; Fig. 6 A). Phylogenetic trees based on heavy- and light-chain nucleotide sequences (GC-Bmem cells: days 9–19, LN3; days 21–31, LN1 and LN2) or only a heavy-chain nucleotide sequence (Act-Bmem cells: days 9–19, LN3; GC-Bmem cells: days 9–19, LN1) indicated that the memory B cells that share a clonal origin with GC cells tend to emerge early in the process of clonal expansion (Fig. 6 B). Thus, the great majority of memory B cells are not found as large expanded clones and do not originate from the dominant clones in contemporaneous GCs.

### GC-Bmem and Act-Bmem antibody affinity

**Figure 7.**
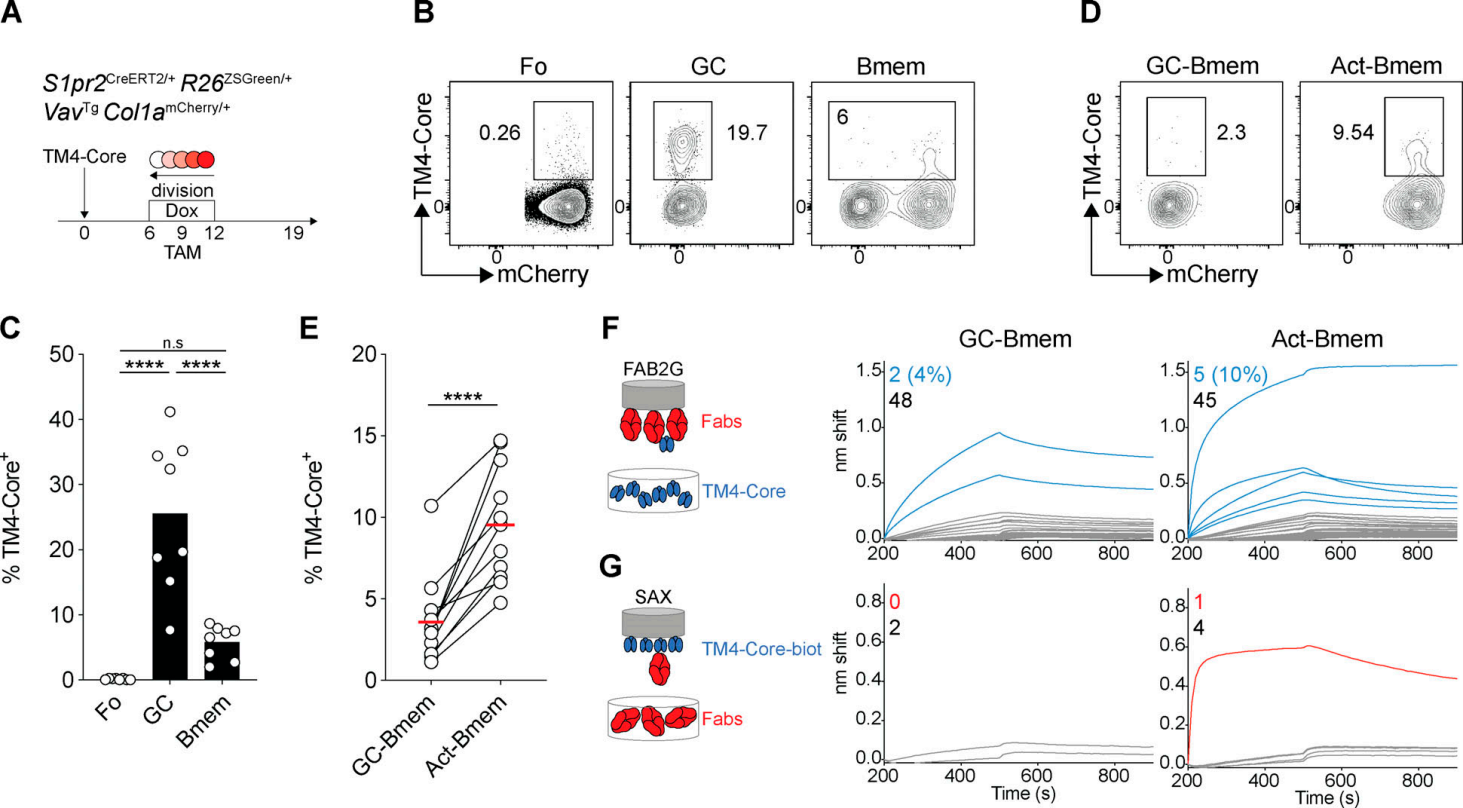
**GC-Bmem and Act-Bmem cell antibody affinity. (A) **Schematic representation of the experiment. **(B and C)** Representative flow cytometry profiles and graph summarizing the data from three independent experiments showing the percentage of TM4-Core–binding cells among follicular (Fo), GC, and memory B cells irrespective of mCherry expression. (Each dot represents one mouse, three independent experiment, *n* = 8.) ****, P ≤ 0.0001) by one-way ANOVA. **(D and E)** Representative flow cytometry profiles and graph showing the percentage of TM4-Core–binding cells among GC-Bmem and Act-Bmem cells in the same mouse. (Each dot represents one mouse, three independent experiments, *n* = 11.) ****, P ≤ 0.0001 by paired *t* test. **(F)** Diagram (left) shows the experimental setup for biolayer interferometry, with biosensor chips loaded with individual Fabs immersed in solutions containing TM4-Core. Graphs (right) show biolayer interferometry traces. Curves in gray represent Fabs whose binding was similar to ED38 negative control Fab. Curves in blue indicate measurable affinity above the negative control. The numbers in blue (positive) and gray (negative) in the upper left of each graph enumerate the Fabs tested. **(G)** As in F, but biosensor chips were loaded with TM4-Core and immersed in solutions containing individual Fabs that showed a significant binding in F. Curves in red indicate when affinity was greater than the negative control. The numbers in red (positive) and gray (negative) in the upper left of each graph enumerate the Fabs tested. Dox, doxycycline; TAM, tamoxifen.

**Figure S5.**
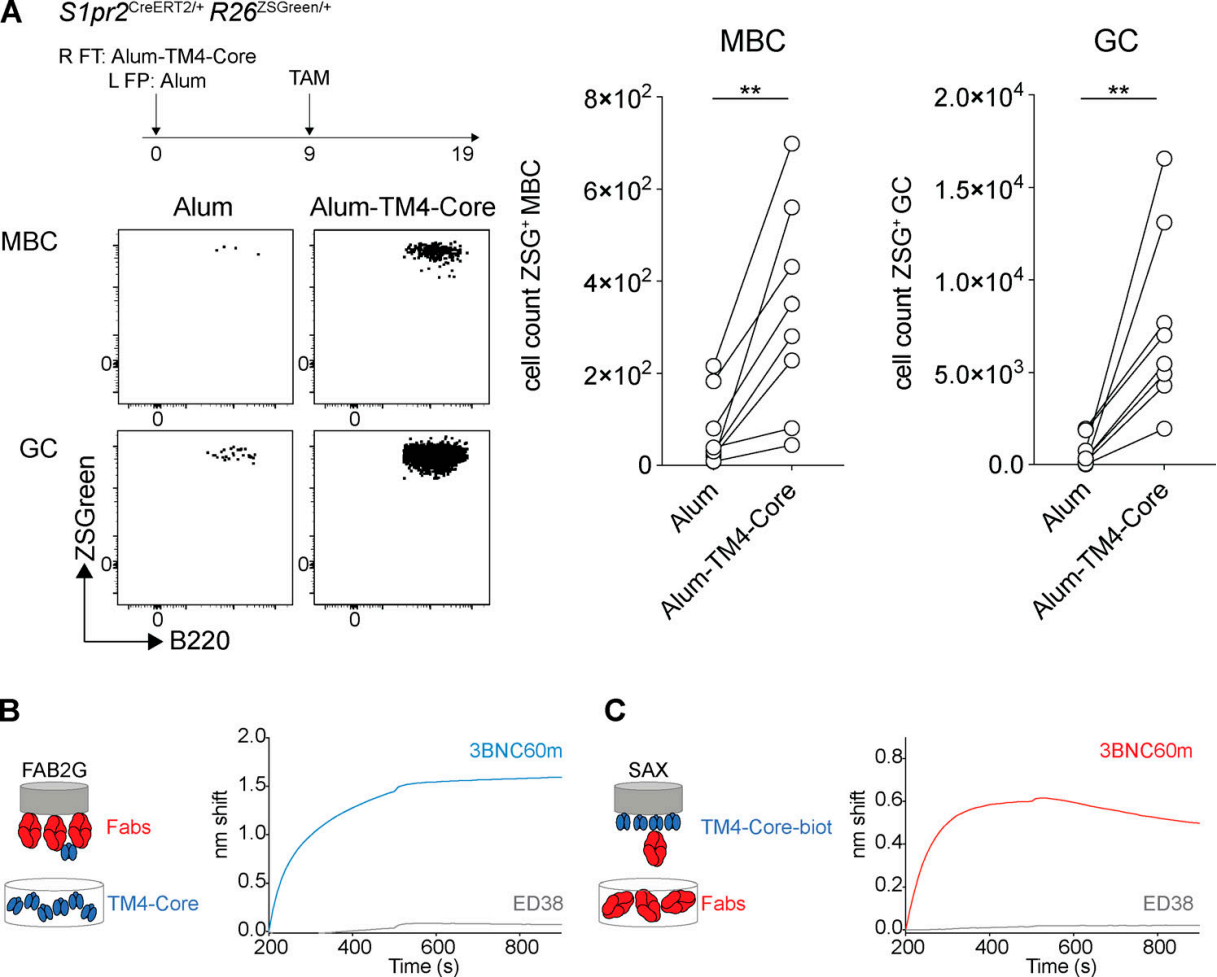
**GC-Bmem and Act-Bmem cell antibody affinity. **Related to Fig. 7. **(A)** Schematic representation of the experiment: *S1pr2*^CreERT2/+^ *R26*^ZSGreen/+^ mice were immunized with HIV-1 alum-TM4-Core (right footpad [R FT]) and alum alone (left footpad [L FT]) on day 0, tamoxifen (TAM) was administered on day 9, and analysis was performed 10 d after tamoxifen administration. Representative flow cytometry profiles and graphs show the number of memory B cells (MBC) and GC cells in the left popliteal lymph node (alum alone) and the right popliteal lymph node (alum-TM4-Core) of the same mice. (Each dot represents one mouse, 3 independent experiments *n* = 8.) **, P ≤ 0.01 by paired *t* test. **(B)** Graphs show biolayer interferometry traces for biosensor chips loaded with individual Fabs immersed in solutions containing TM4-Core. Curves in blue represent the binding of 3BNC60m Fab, the positive control. Curves in gray represent the binding of ED38 Fab, the negative control. **(C)** Graphs show biolayer interferometry traces for biosensor chips loaded with TM4-Core immersed in solutions containing Fabs. Curves in red represent the binding of 3BNC60m Fab, the positive control. Curves in gray represent the binding of ED38 Fab, the negative control. ZSG, ZSGreen.

To examine the antigen-binding properties of the B cell receptors expressed by different types of memory B cells, we immunized double-reporter mice with HIV-1 TM4-Core and treated with doxycycline and then tamoxifen on days 6–12 and 9, respectively. Cells in the draining lymph nodes were assayed for antigen binding by flow cytometry (Fig. 7, A–C). On day 19 after immunization, antigen-binding cells were enriched in the GC compartment compared with resting follicle–origin B cells (P < 0.001; Fig. 7, B and C). As expected, there were many fewer antigen-binding cells in the contemporaneous memory B cell compartment (P = 0.058; Fig. 7, B and C; Viant et al., 2020). GCs and memory B cells were nearly undetectable in mice immunized with adjuvant alone, as a control showed (Fig. S5 A).

To determine the relative proportion of antigen-binding cells in the GC-Bmem and Act-Bmem cell compartments, we separated the two cell types on the basis of their proliferative history. In all 11 mice analyzed, we found a higher proportion of antigen-binding cells among Act-Bmem (9.6%) than GC-Bmem (3.6%, P < 0.001; Fig. 7, D and E) cells.

To document the binding properties of the antibodies expressed by Act-Bmem and GC-Bmem cells, we expressed them as Fabs and performed biolayer interferometry measurements. Memory B cells generally show only low affinity for antigen, which is best revealed by multivalent interactions (Viant et al., 2020). To this end, Fabs were coated on the biosensor to produce a multivalent surface that was then exposed to HIV-1 TM4-Core trimer (Fig. 7 F). 3BNC60, an anti-HIV-1 CD4 binding site–specific antibody, served as a positive control, and ED38, a polyreactive antibody, served as a negative control (Fig. S5 B). Consistent with the flow cytometry data, 2 of 50 GC-Bmem (4%) and 5 of 50 Act-Bmem (10%) cells showed significant binding (Fig. 7 F). Among these seven antibodies, only one Act-Bmem Fab showed significant binding under monovalent conditions when HIV-1 TM4-Core trimer was immobilized on the biosensor chip and subsequently exposed to the cloned Fab (Fig. 7 G and Fig. S5 C). In conclusion, memory B cells with measurable affinity for HIV-1 TM4-Core are more prevalent among Act-Bmem than GC-Bmem cells on day 19 after immunization with TM4-Core.

## Discussion

We documented the emergence of two memory B cell populations in mice with an intact immune system in response to an HIV-1 antigen using an unbiased approach that combines fate mapping and cell division. Act-Bmem and GC-Bmem cells are closely related cell types that differ in gene expression, class-switch recombination, somatic hypermutation, and antigen-binding affinity.

Memory B cell subsets have been defined by isotype (Dogan et al., 2009; Gitlin et al., 2016; Pape et al., 2011) and overall gene expression (Laidlaw et al., 2020) as well as by CD80 and PD-L2 surface expression (Shlomchik, 2018; Tomayko et al., 2010; Zuccarino-Catania et al., 2014). Separating memory B cells based on their origins confirms that CD80^+^PD-L2^+^, Mem α, and Mem β subpopulations originate in the GC. In contrast, memory cells originating from the activated B cell compartment show a distinct pattern of gene expression that does not entirely correspond to previously characterized memory populations but may be closest to CD80^−^PD-L2^−^. Despite their overall transcriptional similarities, the two cell types retain traces of their origins, GC-Bmem cells are closely related to GC B cells, and Act-Bmem cells are closely related to follicular origin B cells. Why Act-Bmem cells are more closely related to follicular origin B cells than their immediate precursors, activated B cells, may be a function of the heterogeneity of the latter compartment, which also gives rise to plasmablast and GC cells. The combination of lineage tracking and cell division history clarifies some of the distinctions and helps to delineate the two cell types.

Memory B cell subpopulations have documented functional differences. For example, IgM^+^ memory B cells are longer lived than isotype-switched memory B cells that carry higher numbers of somatic mutations as the CD80^+^ memory B cells (Dogan et al., 2009; Pape et al., 2018; Pape et al., 2011). The mutations can increase polyreactivity (Tiller et al., 2007), which is associated with accelerated elimination of isotype-switched memory cells (Gitlin et al., 2016). IgM^+^ memory B cells and CD80^−^ memory B cells preferentially participate in secondary GCs upon secondary immunization (Dogan et al., 2009; Gitlin et al., 2016; Pape et al., 2011; Zuccarino-Catania et al., 2014). In contrast, CD80^+^, PD-L2^+^IgM^+^, and all IgG^+^ memory B cells generate an early burst of antibody-forming cells upon secondary immunization (Krishnamurty et al., 2016; Zuccarino-Catania et al., 2014). Our observation that Act-Bmem cells, which are non–GC-origin IgM^+^CD80^−^ cells, carry a greater fraction of higher-affinity receptors than GC-Bmem cells may explain why cells with this phenotype are favored to reenter secondary GCs.

After an encounter with antigen, activated B cells migrate to the T–B border where they can differentiate into GC cells, plasmablasts, or Act-Bmem cells (Harwood and Batista, 2010; Vinuesa et al., 2016; Lau and Brink, 2020; Laidlaw and Cyster, 2021). To date, Act-Bmem cells were thought to be produced early in the immune response, before GCs coalesce, and supplanted thereafter by GC-Bmem cells (Blink et al., 2005; Pape et al., 2011; Taylor et al., 2012; Weisel et al., 2016). Our experiments indicate that activated B cells continue to differentiate into Act-Bmem cells during the entire immune response. This observation is consistent with the idea that the activated B cell compartment persists throughout the immune response and that high-affinity cells in this compartment can continue to join GCs (Schwickert et al., 2009).

The decision by an activated B cell to differentiate into a plasmablast, GC cell, or Act-Bmem cell is influenced in large measure by antigen-binding affinity with a bias for higher-affinity cells developing into plasma and GC cells (Smith et al., 2000; Chan et al., 2009; Victora et al., 2010; Gitlin et al., 2014; Taylor et al., 2015; Kräutler et al., 2017). The decision to enter the Act-Bmem compartment is likely to occur early, before class-switch recombination, extensive clonal expansion, or hypermutation, because Act-Bmem cells express IgM and IgD, are not found in large expanded clones, and carry few somatic mutations (Roco et al., 2019). In addition to affinity as a driver for differentiation, secondary isotype expression could disfavor Act-Bmem cell formation because of differences in signaling by the two different types of B cell receptors (Gitlin et al., 2016; Horikawa et al., 2007; Martin and Goodnow, 2002).

GC-Bmem cell differentiation is also influenced by antigen binding. GC-Bmem cells are cells whose relative antigen-binding activity is lower than competing cells in the GC (Viant et al., 2020), leading to increased CCR6 (Suan et al., 2017), Ephrin-B1 (Laidlaw et al., 2017), Bach-2 (Shinnakasu et al., 2016), Tel3, and Hhex (Laidlaw et al., 2020) and decreased Bcl6 (Wang et al., 2017) expression. Consistent with their lower relative affinity, we find that GC-Bmem precursors express antibodies that typically fail to support large clonal expansion in the GC.

In conclusion, differentiation into the two memory B cell subsets occurs throughout the immune response to HIV-1 TM4-Core. Act-Bmem and GC-Bmem cells differ in gene expression in a manner consistent with their origins from activated and GC B cells. Notably, the fraction of high-affinity antigen-binding B cells in the Act-Bmem compartment is higher than GC-Bmem cells, which may account for the preferential participation of cells with this phenotype in secondary immune responses.

## Materials and methods

### Mice

*Vav*^Tg^ *Col1a*^mCherry/+^ mice were described in Gitlin et al. (2014), *S1pr2*-ERT2cre mice were provided by T. Kurosaki (Laboratory of Lymphocyte Differentiation, WPI Immunology Frontier Research Center, Osaka University, Osaka, Japan; Shinnakasu et al., 2016), and *Rosa*-ZGreen (Ai6; Rosa-CAG-LSL-ZsGreen1-WPRE; Stock No. 007914) mice were purchased from The Jackson Laboratory. All mutations were obtained and maintained on a C57BL/6J background. All animal procedures were performed in accordance with protocols approved by the Rockefeller University institutional animal care and use committee.

### Immunizations and tamoxifen or anti-CD40L injections

Footpad immunizations were performed with 25 µl of PBS containing 5 µg of HIV envelope antigen TM4-Core provided by Andrew T. McGuire (Fred Hutchinson Cancer Research Center, Seattle, WA) and L. Stamatatos and precipitated in alum (Imject Alum; Thermo Fisher Scientific) at a 2:1 ratio. Activation of the Cre recombinase in the *S1pr2*-ERT2cre mice was induced by one oral administration of 12 mg tamoxifen (T5648; Sigma) in 200 µl of corn oil (C8267; Sigma) at the indicated time points. mCherry dilution was initiated by intraperitoneal injection of 2 mg doxycycline (D9891; Sigma) in 1× PBS followed by supplementing the drinking water for the next 6 d with 1 g/liter doxycycline and 5% sucrose (S0389; Sigma). Depletion of GC cells was achieved by i.v. injection of CD40L every 3 d since day 20 to day 42 (300 µg in 1× PBS, BE0017-1; Bio X Cell).

### Flow cytometry

Lymph nodes (axillary or popliteal) were collected in FACS buffer (1× PBS, 10% FCS, 2 mM EDTA) on ice. Single-cell suspensions were obtained by mechanical disruption through a 70-mm cell strainer (BD Biosciences). Erythrocytes were lysed with 1 ml ACK lysing buffer (Gibco). After incubation with 5 µg/ml anti-CD16/32 (rat mAb 2.4G2, mouse Fc block; BD Biosciences) for 15 min at 4°C, cells were stained for 30 min at 4°C. When a biotinylated antibody was used, the cells were then incubated with a streptavidin–fluorophore conjugate for 20 min at 4°C. Flow cytometric analysis was performed on a BD LSRFortessa and Symphony. Antibodies used: from BD Biosciences, anti-IgM-e710 (R6-60.2, 550881), anti-IgD-BV786 (11-26c.2a, 563618), anti-CD95-PE-Cy7 (Jo2, 557653), and streptavidin-BV711 (563262); from BioLegend, anti-CD38-PB (90, 102719), anti-B220-BV605 (RA3-6B2, 103244), and live/dead marker Zombie NIR (423106); and from eBiosciences, anti-T and -B cell activation antigen-e660 (GL7, 50-5902-82), anti-CD4-eF780 (RM4-5, 47-0042-82), anti-CD8-eF780 (53-6.7, 47-0081-82), anti-NK1.1-eF780 (PK136, 47-5941-82), anti-F4/80-eF780 (BM8, 47-4801-82), and anti-TM4-Core-biot (5 µg/ml) provided by Andrew T. McGuire and L. Stamatatos.

### RNA-seq

Cells were sorted directly into a solution of 1% 2-β-mercaptoethanol (Sigma) in TCL Buffer (1031576; QIAGEN), and RNA was isolated using RNAClean XP Beads (A63987; Beckman Coulter). Near full-length mRNA was reverse transcribed as previously described (Islam et al., 2014; Trombetta et al., 2014). Multiplexed libraries were prepared for sequencing using a Nextera XT DNA Library Preparation Kit (Illumina). Libraries were sequenced on an Illumina NovaSeq 6000 at the Rockefeller University Genomics Core.

Sequence reads were pseudo-aligned to an index created from the Ensembl mouse GRCm38.p5 assembly. Transcript-level abundances were quantified using kallisto v0.44.0 (Bray et al., 2016) and subsequently summarized to gene level using the R package tximport (Soneson et al., 2015). Counts normalization and differential gene expression analysis were performed using DESeq2 (Love et al., 2014). Data files for the RNA-seq analyses have been deposited in the National Center for Biotechnology Information Gene Expression Omnibus under accession no. GSE174394.

### Bulk RNA-seq and single-cell RNA-seq comparison

Using Seurat v3.1.2, we reanalyzed the single-cell data from Laidlaw et al. (2020). Cells containing >35% of mitochondrial DNA were filtered out, and a population of 1,512 memory B cells was defined using the cell markers Zeb2 and CD38 expression. This population was further subdivided in 784 Mem α cells, 490 Mem β cells, and 238 prememory B cells according to Mki67, Bcl6, and Bcl2 expression. To simulate a bulk RNA-seq experiment, we summed up the reads for each gene across all cells for each B cell subpopulation. Shared genes between the simulated bulk RNA-seq and the authentic bulk RNA-seq were loaded into a single expression matrix. The R package sva (v3.36.0) was used to correct the batch effect before principal component analysis using normalized counts calculated by DeSeq2 v1.28.1. Data files for the RNA-seq analyses have been deposited in the National Center for Biotechnology Information Gene Expression Omnibus under accession no. GSE174394 and files for the single-cell RNA-seq (Laidlaw et al., 2020) are available under the accession no. GSE148805.

### Single-cell index sorting and RNA purification

B cells from draining lymph nodes were negatively enriched or not with CD43 (Ly-48) MicroBeads (130-049-801; Miltenyi Biotec), stained, single-cell sorted with a FACSAria II (BD Biosciences) in 96-well plates containing 5 µl of a lysis buffer (TCL Buffer, 1031576; QIAGEN) containing 1% 2-β-mercaptoethanol (M3148; Sigma) and immediately frozen at −80°C. RNA was purified from single cells using magnetic beads (RNAClean XP, A63987; Beckman Coulter) following the manufacturer’s instructions. RNA was eluted from the magnetic beads with 11 µl of a solution containing random primers (14.5 ng/µl, 48190-011; Invitrogen), tergitol (0.5% NP-40 70% in H_2_O, NP-40S; Sigma-Aldrich), and RNase inhibitor (0.6 U/µl, N2615; Promega) in nuclease-free water (QIAGEN) and incubated at 65°C for 3 min. cDNA was subsequently synthesized by reverse transcription with 7 µl of a solution containing SuperScript III Reverse Transcription, 5× buffer, dithiothreitol (10,000 U, 18080-044; Invitrogen), dNTP (25 µM), and RNase inhibitor (0.6 U/µl, N2615; Promega) in nuclease-free water (QIAGEN) incubated at 1× (42°C for 10 min, 25°C for 10 min, 50°C for 60 min, 94°C for 5 min). cDNA was stored at −20°C or immediately used for antibody gene amplification by nested PCR after addition of 10 µl nuclease-free water.

### Antibody sequencing and cloning

Mouse antibody genes were amplified by nested PCR using 42 µl of a solution containing Hot Star Taq DNA polymerase (250 U/50 µl) and 10× buffer (203209; QIAGEN), dNTP (25 µM), 5′ forward primers (50 µM), 3′ reverse primers (50 µM), and 4 µl cDNA for PCR1 or PCR1 product for PCR2 in nuclease-free water (QIAGEN; von Boehmer et al., 2016). PCR1 protocol was heavy chain (IgM and IgG) and light chain (IgK) 1× (95°C for 15 min), 50× (94°C for 30 s, 46°C for 30 s, and 72°C for 55 s), and 1× (72°C for 10 min). PCR2 protocol was light chain using the same protocol as PCR1 and heavy chain using the same protocol except for the annealing temperature (55°C).

Cloning PCR protocol was 1× (95°C for 15 min), 50× (94°C for 30 s, 50°C for 30 s, 72°C for 55 s), and 1× (72°C for 10 min). Amplified cDNA was purified with QIAquick 96 PCR Purification Kit (QIAGEN) following the manufacturer’s instructions. The linearized human Fab vector (30–50 ng) and insert (4 µl of purified product) were ligated at 25°C for 2.5 min. Ligation was transformed in DH5α-competent bacteria. The next day, bacterial colonies were analyzed by PCR. PCR protocol was 1× (95°C for 15 min), 50× (94°C for 30 s, 57°C for 30 s, 72°C for 55 s), and 1× (72°C for 10 min).

### Fab expression

Ig sequences were cloned into human IgH Fab and IgK plasmids, and Fabs were expressed by transient transfection of HEK293-6E cells and purified with Ni Sepharose 6 Fast Flow (GE Healthcare). After buffer exchange in PBS, the yield was determined by measurement with nanodrop analysis and PAGE.

### Fab affinity and avidity analysis

Biolayer interferometry assays were performed on the Octet RED instrument (FortéBio) at 30°C with shaking at 1,000 rpm. For affinity measurements, all measurements of HIV-1 TM4-Core/Fab binding were corrected by subtracting the signal obtained from measurements performed with HIV-1 TM4-Core but in the absence of Fabs. The same trimer used for the immunization was biotinylated using EZ-Link NHS-PEG4-Biotin (21330; Thermo Fisher Scientific). Biotinylation was performed by adding biotin at a 1:1 ratio with the Env trimer, and unligated biotin was removed using Zebra desalting columns (21330; Thermo Fisher Scientific). The kinetic analysis using high-precision streptavidin biosensor (18-5118; FortéBio) was performed as follows: (1) baseline: 60-s immersion in buffer (kinetics buffer 10×, 18-1105; FortéBio); (2) loading: 200-s immersion in a solution with biotinylated trimeric TM4-Core at 400 nM; (3) baseline: 200-s immersion in buffer; (4) association: 300-s immersion in solution with Fab 10 µg/ml; and (5) dissociation: 600-s immersion in buffer. Curve fitting was performed using a fast 2:1 binding model and data analysis software (FortéBio). For measurement at increased valency, all measurements of Fab/TM4-Core binding were corrected by subtracting the signal obtained from measurements performed with the same Fab but in absence of TM4-Core. The kinetic analysis using FAB2G biosensor (18-5125; FortéBio) was performed as explained above for the affinity measurements. Curve fitting was performed using a bivalent model and data analysis software (FortéBio).

### Statistical analyses

Statistical information, including *n*, mean, and statistical significance values, is indicated in the figure legends. Statistical significance was determined with GraphPad Prism 7 using the tests indicated in each figure. Data were considered statistically significant at *, P ≤ 0.05; **, P ≤ 0.01; ***, P ≤ 0.001; and ****, P ≤ 0.0001.

### Online supplemental material

Fig. S1 explains how H2B-mCherry mice can be used to track proliferation and shows the gating strategy used to identify B cell populations. Fig. S2 shows depletion of GC cells after repeated injections of CD40L blocking antibody. Fig. S3 illustrates the sorting strategy used for the RNA-seq analysis of B cell populations and shows the hierarchical-clustering heat maps of genes involved in the GSEA, revealing that significant gene expression increases in GC-Bmem cells compared with Act-Bmem cells in several pathways (Fig. 3). Fig. S4 shows that activated B cells can accumulate somatic mutations 3 d after immunization, before GC formation. It documents the level of *Aicda* expression in different B cells populations, the single-cell sorting strategy for activated B cells 3 d after immunization, and the number of somatic mutations found in these cells. Fig. S5 shows that immunization with alum alone fails to elicit GC and memory B cells. It also shows the binding of 3BNC60m Fab-positive control and ED38 Fab-negative control to TM4-Core by biolayer interferometry assays. Table S1 describes the expression of genes enriched in CD80^+^PD-L2^+^ memory B cells. Table S2 describes the expression of genes enriched in CD80^−^PD-L2^−^ memory B cells.

## Supplementary Material

Table S1 describes the expression of genes enriched in CD80^+^PD-L2^+^ memory B cells (related to Fig. 4).

Table S2 describes the expression of genes enriched in CD80^−^PD-L2^−^ memory B cells (related to Fig. 4).
