# Correction: Germinal center–dependent and –independent memory B cells produced throughout the immune response

**DOI:** 10.1084/jem.2020248904162024c

**Published:** 2024-04-29

**Authors:** Charlotte Viant, Tobias Wirthmiller, Mohamed A. ElTanbouly, Spencer T. Chen, Ervin E. Kara, Melissa Cipolla, Victor Ramos, Thiago Y. Oliveira, Leonidas Stamatatos, Michel C. Nussenzweig

Vol. 218, No. 8 | https://doi.org/10.1084/jem.20202489 | June 9, 2021

In the original version of this article, Dr. Ervin E. Kara was omitted from the author list. Dr. Kara had helped establish the direction of the project but left the laboratory two years before the manuscript was submitted to *JEM*. During preparation of the manuscript, Dr. Kara was asked whether he would like to be an author, but he was unavailable to participate. He has since become available and is now included as an author. All co-authors have approved this addition. The corrected author list appears above. The error appears in print and in PDFs downloaded before April 16, 2024.

