# Supplementary material for: Germinal center–dependent and –independent memory B cells produced throughout the immune response
Source: J Exp Med. 2021 Jun 9;218(8):e20202489. doi: 10.1084/jem.20202489 (PMC8193567; doi:10.1084/jem.20202489)
Supplement: Table S1 — describes the expression of genes enriched in CD80+PD-L2+ memory B cells (related to Fig. 4). [file JEM_20202489_TableS1.docx]

**Table S1. Expression of genes enriched in CD80^+^PD-L2^+^ memory B cells**

| CD80^+^PD-L2^+^ > CD80^-^PD-L2^-^ | | | |
| --- | --- | --- | --- |
|  | Act-Bmem | GC-Bmem | q-value |
| CCND2 | 2028 | 3544 | 0.16 |
| PDLIM1 | 407 | 1682 | 0.01 |
| ASS1 | 471 | 2599 | 3.57E-05 |
| CKB | 26 | 136 | 0.05 |
| VIM | 5912 | 17958 | 3.36E-13 |
| MYADM | 191 | 675 | 1.36E-08 |
| SGK1 | 172 | 133 | 0.46 |
| AHNAK | 421 | 1274 | 0.22 |
| RASSF4 | 633 | 1520 | 0.24 |
| ANXA2 | 2875 | 5700 | 0.03 |
| CD86 | 1133 | 1481 | 0.54 |
| SLPI | 1431 | 5516 | 0.06 |
| ZBTB32 | 173 | 1550 | 0.00 |
| SEMA4F | 37 | 34 | 0.92 |
| FSCN1 | 26 | 143 | 0.01 |
| S100A6 | 165 | 254 | 0.77 |
| CD80 | 52 | 408 | 7.32E-09 |
| P2RY13 | 3 | 71 | 0.02 |
| SIAT7C | 322 | 147 | 0.23 |
| CCBP2 | 61 | 547 | 0.00 |
| 9130213B05RIK | 236 | 1452 | 5.37E-05 |
| ZFP288 | 3220 | 7496 | 5.82E-07 |
| KLF2 | 1126 | 1510 | 0.36 |
| EMP3 | 1431 | 1028 | 0.13 |

Expression in Act-Bmem and GC-Bmem cells of 24 genes enriched in CD80^+^PD-L2^+^ memory B cells. Font color is red when the expression is significantly different between Act-Bmem and GC-Bmem cells (q-value ≤0.05). Text is highlighted in yellow when the expression is significantly different and higher in GC-Bmem cells.
