# Supplementary material for: Germinal center–dependent and –independent memory B cells produced throughout the immune response
Source: J Exp Med. 2021 Jun 9;218(8):e20202489. doi: 10.1084/jem.20202489 (PMC8193567; doi:10.1084/jem.20202489)
Supplement: Table S2 — describes the expression of genes enriched in CD80−PD-L2− memory B cells (related to Fig. 4). [file JEM_20202489_TableS2.docx]

**Table S2. Expression of genes enriched in CD80^-^PD-L2^-^ memory B cells (related to Fig. 4)**

| CD80^+^PD-L2^+^ < CD80^-^PD-L2 | | | |
| --- | --- | --- | --- |
|  | Act-Bmem | GC-Bmem | q-value |
| FER1L3 | 61 | 52 | 0.88 |
| MEF2B | 1447 | 2669 | 0.29 |
| UHRF1 | 424 | 898 | 0.05 |
| EDG5 | 1182 | 1735 | 0.51 |
| CSRP2 | 381 | 368 | 0.98 |
| LRRK2 | 2233 | 1080 | 0.03 |
| BIRC5 | 225 | 1630 | 2.94E-15 |
| PBK | 115 | 233 | 0.27 |
| MKI67 | 795 | 1508 | 0.32 |
| 1190002H23RIK | 114 | 273 | 0.24 |
| DPP4 | 772 | 321 | 0.02 |
| A930005H10R | 217 | 188 | 0.78 |
| SLA | 1571 | 1263 | 0.75 |
| KCNK5 | 122 | 310 | 0.20 |
| MCM5 | 2566 | 2040 | 0.71 |
| SH2D2A | 304 | 275 | 0.93 |
| HIST1H2AF | 15 | 146 | 8.19E-08 |
| HIST1H2AD | 39 | 106 | 0.03 |
| HIST1H2AH | 5 | 11 | 0.57 |
| CD72 | 8701 | 6737 | 0.19 |
| BCL6 | 274 | 171 | 0.36 |
| EPHX1 | 1306 | 1051 | 0.80 |
| SATB1 | 3095 | 562 | 4.02E-21 |
| BLVRB | 1968 | 2868 | 0.12 |
| KLF2 | 1126 | 1510 | 0.36 |
| EMP3 | 1431 | 1028 | 0.13 |
| MYL4 | 358 | 482 | 0.67 |
| KLHL6 | 3527 | 4576 | 0.18 |
| HIST1H2AO | 12 | 25 | 0.18 |
| EG667728 | 0.4 | 1.9 | 0.17 |

Expression in Act-Bmem and GC-Bmem cells of 30 genes enriched in CD80^−^PD-L2^−^ memory B cells. Font color is red when the expression is significantly different between Act-Bmem and GC-Bmem cells (q-value ≤0.05). Text is highlighted in yellow when the expression is significantly different and higher in Act-Bmem cells.
